# The Triggering Receptor Expressed on Myeloid Cells 2 Inhibits Complement Component 1q Effector Mechanisms and Exerts Detrimental Effects during Pneumococcal Pneumonia

**DOI:** 10.1371/journal.ppat.1004167

**Published:** 2014-06-12

**Authors:** Omar Sharif, Riem Gawish, Joanna M. Warszawska, Rui Martins, Karin Lakovits, Anastasiya Hladik, Bianca Doninger, Julia Brunner, Ana Korosec, Roland E. Schwarzenbacher, Tiina Berg, Robert Kralovics, Jacques Colinge, Ildiko Mesteri, Susan Gilfillan, Andrea Salmaggi, Admar Verschoor, Marco Colonna, Sylvia Knapp

**Affiliations:** 1 CeMM - Research Center for Molecular Medicine of the Austrian Academy of Sciences, Vienna, Austria; 2 Department of Medicine I, Laboratory of Infection Biology, Medical University of Vienna, Vienna, Austria; 3 Department of Pathology, Medical University of Vienna, Vienna, Austria; 4 Department of Pathology and Immunology, Washington University School of Medicine, St. Louis, Missouri, United States of America; 5 Department of Clinical Neurosciences, Istituto Nazionale Neurologico Carlo Besta, Milano, Italy; 6 Institute for Medical Microbiology, Immunology and Hygiene, Technical University Munich, Munich, Germany; The University of Texas Health Science Center at San Antonio, United States of America

## Abstract

Phagocytosis and inflammation within the lungs is crucial for host defense during bacterial pneumonia. Triggering receptor expressed on myeloid cells (TREM)-2 was proposed to negatively regulate TLR-mediated responses and enhance phagocytosis by macrophages, but the role of TREM-2 in respiratory tract infections is unknown. Here, we established the presence of TREM-2 on alveolar macrophages (AM) and explored the function of TREM-2 in the innate immune response to pneumococcal infection *in vivo*. Unexpectedly, we found *Trem-2* ^−/−^ AM to display augmented bacterial phagocytosis in vitro and in vivo compared to WT AM. Mechanistically, we detected that in the absence of TREM-2, pulmonary macrophages selectively produced elevated complement component 1q (*C1q*) levels. We found that these increased C1q levels depended on peroxisome proliferator-activated receptor-δ (PPAR-δ) activity and were responsible for the enhanced phagocytosis of bacteria. Upon infection with *S. pneumoniae*, *Trem-2* ^−/−^ mice exhibited an augmented bacterial clearance from lungs, decreased bacteremia and improved survival compared to their WT counterparts. This work is the first to disclose a role for TREM-2 in clinically relevant respiratory tract infections and demonstrates a previously unknown link between TREM-2 and opsonin production within the lungs.

## Author Summary

Bacterial respiratory tract infections are a major cause of morbidity and mortality, and *Streptococcus pneumoniae* (*S. pneumoniae*) remains the main cause of community acquired pneumonia worldwide. The continued rise in antibiotic resistance stresses the need for better insights into the host defense mechanisms associated with pneumococcal pneumonia. The early innate immune response that constitutes bacterial phagocytosis, complement activation and inflammation is critical for the outcome during pneumonia. The triggering receptor expressed on myeloid cells 2 (TREM-2) has recently been shown to be both a negative regulator of the inflammatory response and a promoter of phagocytosis, but its contribution to pneumonia remains unknown. In our study, we unexpectedly found that alveolar macrophage expressed TREM-2 is detrimental in bacterial phagocytosis and clearance during pneumococcal pneumonia. This occurred via the suppressive effects of TREM-2 on complement component 1q (C1q), an important regulator of bacterial phagocytosis that is crucial for the host response during pneumonia. Thus, targeting the TREM-2 pathway could be used as a novel strategy for modulating C1q production and pulmonary innate immune responses, which could be of clinical relevance during pneumonia and other respiratory tract infections.

## Introduction

Phagocytosis is the process by which cells ingest particles and is a major effector mechanism of the innate immune system. Professional phagocytes such as macrophages use a variety of surface receptors including scavenger-, Fc- and opsonin-receptors to internalize microbes. In addition, innate immune receptors such as Toll like receptors (TLRs) recognize conserved microbial structures and trigger the production of pro-inflammatory cytokines and chemokines, thereby shaping both the innate and adaptive immune response.

TLR activation and phagocytosis are intimately linked. Upon phagocytosis of Gram positive and negative bacteria, TLR2 and 4 initially located at the plasma membrane accumulate into phagosomes, sample their contents and elicit immune responses to products which only become accessible after digestion of the cell wall of bacteria [1], [2], [3]. TLR activation also enhances the expression of phagocytic receptors such as scavenger receptor A or MARCO and elevates opsonin levels [4], [5], [6], [7]. The prime aim of such inflammatory phagocytosis is to urgently remove invading pathogens before they multiply, invade other tissues and spread systemically. The rapid elimination of pathogens thus prevents excessive inflammation that can otherwise result in immunopathology and organ failure. Understanding the mechanisms that control phagocytosis and limit inflammation is important as this has implications in the survival from infectious diseases. The triggering receptor expressed on myeloid cells 2 (TREM-2) has been proposed to regulate both processes.

TREM-2 belongs to a conserved but functionally distinct gene family of proteins, with the best studied family members including TREM-1 and 2 [8], [9]. TREM-2 is a receptor with an unknown ligand and is expressed by several cell types including bone marrow derived macrophages (BMDM), microglia and osteoclasts [10], [11], [12]. Humans with mutations in TREM-2 develop Nasu-Hakola disease, which is characterized by progressive dementia and bone cysts [12], [13]. Furthermore, recent evidence shows individuals who possess heterozygous rare variants of TREM-2 are at increased risk of Alzheimer's disease [14], [15]. TREM-2 signals via the immunoreceptor tyrosine-based activation motif (ITAM) of the adaptor protein DNAX activation protein of 12 kDa (DAP-12) to mediate its downstream effects. DAP-12 is rather promiscuous and is used by many other receptors having both activating and inhibitory functions [16]. When TREM-1 is engaged, it potentiates the immune response to bacteria and TLR ligands [17], [18], [19]. Conversely, TREM-2 was reported to function as a negative regulator of TLR mediated inflammation [10], [11], [20]. Therefore, TREM family members act as fine tuners of TLR mediated innate immune responses. Significantly, TREM-2 also plays an important role in phagocytosis. Over-expression of TREM-2 in Chinese hamster ovary (CHO) cells confers binding of both *Staphylococcus aureus* and *Escherichia coli* and TREM-2 mediates phagocytosis of *E. coli* in BMDM [21]. The in vivo relevance of the phagocytic capacity of TREM-2 in relation to *E. coli* peritonitis has been corroborated by a recent study showing that administration of bone marrow cells that over-express TREM-2 enhances bacterial clearance and improves survival in a cecal ligation and puncture (CLP) model [22].

In this report we examine the function of TREM-2 in the context of *Streptococcus pneumoniae* infection, the most frequent cause of community acquired pneumonia [23]. We chose to examine TREM-2 in this context for several reasons. Firstly, TREM-2 is expressed on human AM as well as mouse bronchial epithelial cells, suggesting a role for TREM-2 within the pulmonary compartment [24], [25]. Secondly, pulmonary expression of TREM-2 and DAP-12 increases in mice following *Mycobacterium bovis* infection [26], but the functional importance of TREM-2 in lung host defense is unknown. Lastly, both the early innate immune response and phagocytosis are critical for the outcome during pneumococcal pneumonia [27], [28], [29], [30]. Thus, we hypothesized that studying TREM-2 in the context of pneumococcal pneumonia would provide an ideal model system for examining the cross-talk between TLR signaling and phagocytosis within the lungs.

## Results

### Pulmonary TREM-2 expression and function during early pneumococcal pneumonia

**Figure 1:**
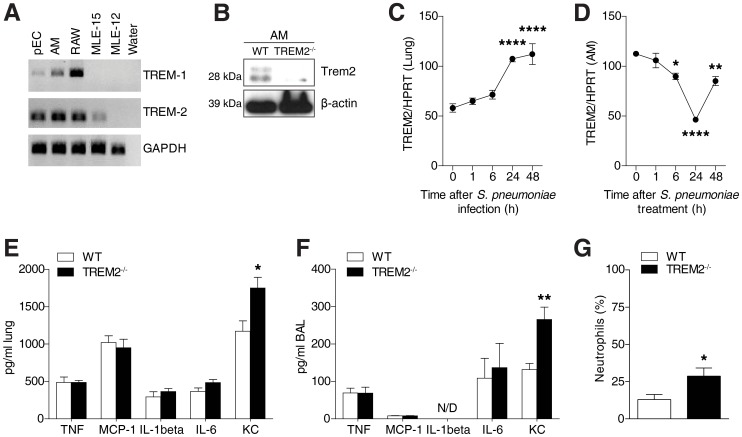
Pulmonary TREM-2 expression and function during *S. pneumoniae* induced inflammation. (**A**) TREM-1 and TREM-2 expression was evaluated in the indicated cell types using RT-PCR. (**B**) Western blot was used to evaluate TREM-2 expression on AM. (**C**) WT mice (n = 6 per time point) were intranasally inoculated with 10^5^ CFU *S. pneumoniae* and after indicated time points TREM-2 lung transcript levels were evaluated. (**D**) WT AM were treated with 2×10^7^ CFU/ml *S. pneumoniae* for indicated time points and TREM-2 RT-PCR was conducted. (**E–G**) WT and *Trem-2* ^−/−^ mice (n = 7 per genotype) were intranasally infected with 10^5^ CFU *S. pneumoniae* for 6 h and levels of indicated cytokines were evaluated in the lung (**E**) or BALF (**F**) and neutrophil counts were determined in the BALF (**G**). Data represent mean ± SEM and are (**A–G**) representative of two independent experiments. Differences were calculated versus time point 0 (**C–D**) or versus WT (**E–G**) and are indicated as * p<0.05, ** p<0.005, **** p<0.0001.

To investigate the role of pulmonary TREM-2 in the context of bacterial pneumonia, we first established which cell types expressed TREM-2 within the lungs. We determined TREM transcript levels in primary AM and respiratory epithelial lung cells (pEC) as well as epithelial cell lines such as alveolar MLE-12 and bronchial MLE-15 cells, respectively [31], and included RAW 264.7 macrophages as a positive control [32], [33]. While TREM-1 expression was predominantly restricted to macrophages, we discovered TREM-2 to be strongly expressed in both AM and respiratory epithelial cells (**Fig. 1A**). We confirmed these results by demonstrating expression of TREM-2 on primary AM using western blot (**Fig. 1B**). Interestingly, we detected two bands in AM, with the upper band probably corresponding to a glycosylated form of TREM-2 as previously observed [34]. Specificity for the antibody was provided as minimal detection of TREM-2 was observed in TREM-2 deficient AM. We then sought to determine pulmonary TREM-2 expression upon *S. pneumoniae* infection and found a time dependent upregulation in whole lung transcript levels, with highest expression 48 h post infection (**Fig. 1C**). This increase in pulmonary TREM-2 expression during infection most likely reflected the influx of TREM-2 expressing cells, since TREM-2 transcript levels on primary AM declined following *S. pneumoniae* treatment (**Fig. 1D**). Together, TREM-2 was abundantly expressed within healthy lungs and further induced upon infection with *S. pneumoniae*.

To exploit the functional role of this constitutive pulmonary TREM-2 expression in relation to early pulmonary inflammatory responses following bacterial infection, we next infected WT and *Trem-2* ^−/−^ mice with *S. pneumoniae* for 6 h. Much to our surprise, since TREM-2 was earlier considered a negative regulator of inflammation [10], [20], we did not identify any differences in levels of several inflammatory mediators tested, such as TNF-α, MCP-1, IL-1β and IL-6 (**Fig. 1E and F**). In fact *Trem-2* ^−/−^ mice only displayed elevated levels of KC, a chemokine required for neutrophil influx following bacterial respiratory tract infections [35] (**Fig. 1E and F**). This was accompanied by a modest increase in recruited neutrophils (**Fig. 1G**). Altogether, these data demonstrate that lung TREM-2 only partially dampened inflammation following *S. pneumoniae* infection in vivo.

### Decreased TLR2 mediated cytokine production in TREM-2 deficient AM

**Figure 2:**
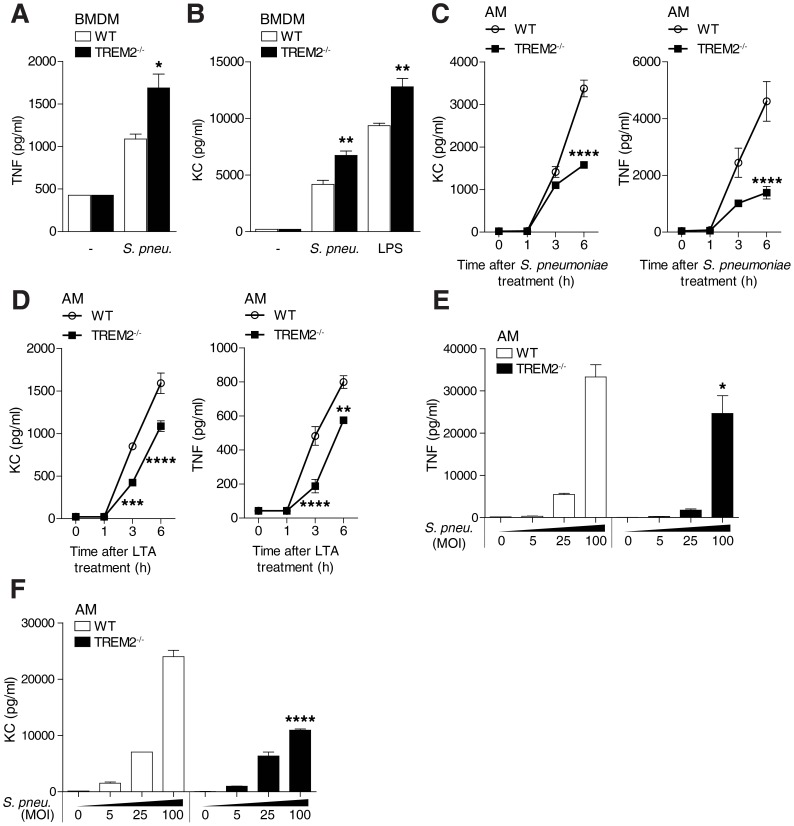
Cell type specific effects of TREM-2 on *S. pneumoniae* and TLR2 mediated cytokine production. (**A–B**) WT and *Trem-2* ^−/−^ BMDM (n = 4 per genotype) were treated with 2×10^7^ CFU/ml *S. pneumoniae* or 100 ng/ml LPS for 6 h and TNF-α (**A**) or KC (**B**) levels were measured in the supernatant. (**C–D**) WT and *Trem-2* ^−/−^ AM (n = 4 per genotype/time point) were treated with 2×10^7^ CFU/ml *S. pneumoniae* (**C**) or 10 µg/ml *S. pneumoniae* LTA (**D**) for the indicated times and KC and TNF-α levels were measured in the supernatant. (**E–F**) WT and *Trem-2* ^−/−^ AM (n = 4 per genotype/MOI) were treated with the indicated doses of *S. pneumoniae* and TNF-α (**E**) and KC (**F**) levels were determined in the supernatant. Statistical comparisons are done versus WT cells (**A–B**) at a given timepoint (**C–D**), or at a particular MOI of *S. pneumoniae* (**E–F**), and indicated as: * p<0.05, ** p<0.005, *** p<0.001, **** p = <0.0001. All data represent mean ± SEM and are representative of two independent experiments.

Given that TREM-2 is considered a negative regulator of inflammation, our observation of unaltered cytokine release in lungs of *Trem-2* ^−/−^ mice following *S. pneumoniae* infection was surprising. We therefore first re-examined the regulatory function of TREM-2 and concentrated on macrophage responses to bacteria. We found *S. pneumoniae* or LPS induced TNF-α and KC synthesis by *Trem2^−/−^* BMDM augmented (**Fig. 2A and B**), supporting previous observations of TREM-2 negatively regulating TLR mediated cytokine synthesis [10].

Getting back to the specific role of TREM-2 within the lungs, we then determined whether *Trem-2* ^−/−^ AM would behave similarly to BMDM in response to *S. pneumoniae* or its lipotechoic acid (LTA), a known TLR2 ligand [36]. Strikingly, *Trem-2* ^−/−^ AM displayed opposite effects to *Trem-2* ^−/−^ BMDM. We discovered that TREM-2 deficient AM exhibited decreased inflammation in response to either stimuli (**Fig. 2C and D**). The mechanism whereby ITAM coupled receptors generate inhibitory or activating signals is not well understood. However, the avidity of receptor ligation and thus ligand density has been described as a potential reason for pro- versus anti-inflammatory responses by ITAM associated receptors [16], [37]. To rule out that the dose of bacteria could alter the effect of TREM-2 on TLR signaling, we stimulated primary AM from WT and *Trem-2* ^−/−^ mice with increasing doses of *S. pneumoniae*. While TNF- α and KC release was not different between WT and *Trem-2* ^−/−^ AM at an MOI of 25, synthesis was significantly lower in *Trem-2* ^−/−^ AM stimulated with an MOI of 100 *S. pneumoniae* (**Fig. 2E and F**). These data suggest that TREM-2 regulated TLR2 mediated cytokine synthesis in a cell type specific manner, i.e. it diminished inflammation in BMDM, but partially enhanced it in AM. Furthermore, given that BMDM and AM were stimulated with an identical dose of bacteria, these data indicate that the cell type specific effects of TREM-2 cannot be explained by differences in receptor ligation and avidity.

### Increased phagocytic activity in TREM-2 deficient alveolar macrophages *in vitro and in vivo*

**Figure 3:**
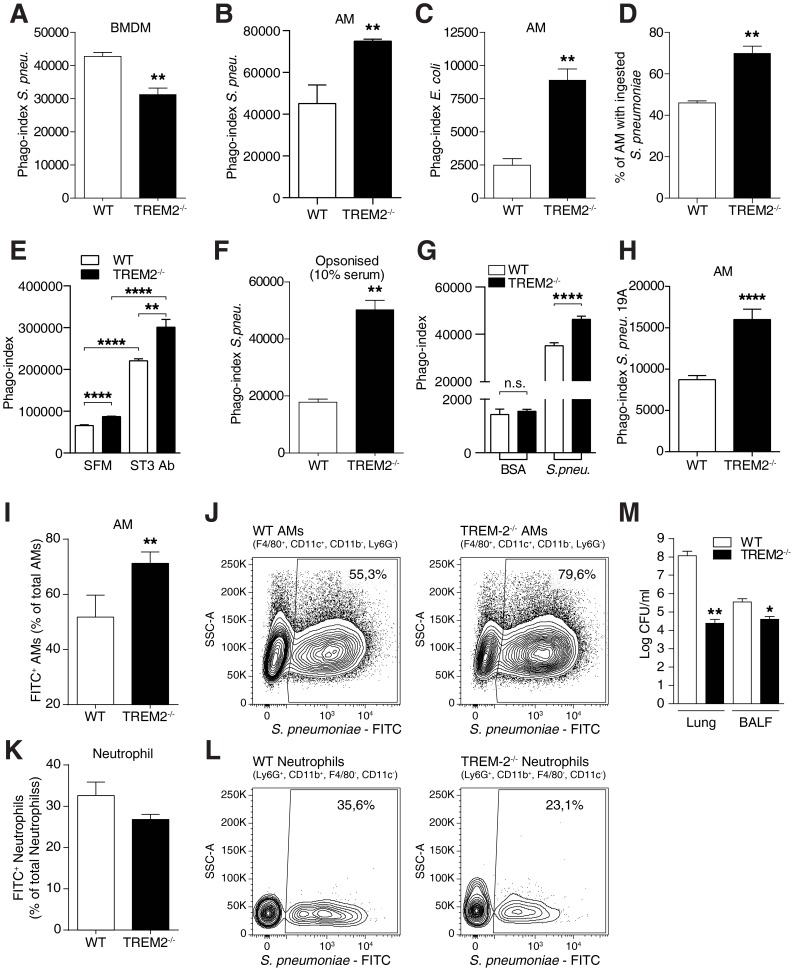
Elevated phagocytosis of bacteria by TREM-2 deficient AM. (**A**) WT and *Trem-2* ^−/−^ BMDM (n = 4–5 per genotype) were incubated with FITC labeled *S. pneumoniae* (MOI 100) and after 1 h phagocytosis was assessed using FACS. (**B–C**) WT and *Trem-2* ^−/−^ AM (n = 4 per genotype) were incubated with FITC labeled *S. pneumoniae* (**B**) or *E. coli* (**C**) (MOI of 100) and phagocytosis was assessed using FACS 1 h later. (**D**) Elevated phagocytosis of *S. pneumoniae* by *Trem-2* ^−/−^ AM as determined using confocal microscopy as described in the M&M section. The percentage of cells that contain bacteria is depicted (n = 4–5 per genotype). (**E–F**) WT and *Trem-2* ^−/−^ AM (n = 4–5 per genotype) were incubated with FITC labeled *S. pneumoniae* (MOI 100) under either serum free conditions (SFM) or the bacteria were pre-opsonised with 10% anti-pneumococcal serotype III capsular antibody (ST3-Ab) (**E**) or 10% pooled WT mouse serum (**F**) for 30 min before addition to the cells. Phagocytosis was assessed 1 h later. (**G**) WT and *Trem-2* ^−/−^ AM (n = 4 per genotype) were incubated with 1 µg/ml FITC labeled BSA or FITC labeled *S. pneumoniae* (MOI 100) and phagocytosis was assessed 1 h later by FACS. (**H**) WT and *Trem-2* ^−/−^ AM (n = 4 per genotype) were incubated with FITC labeled *S. pneumoniae* strain 19A (MOI 100) and phagocytosis was assessed 1 h later by FACS. (**I–L**) WT and *Trem-2* ^−/−^ mice (n = 7 mice per genotype) were intranasally infected with 10^6^ CFU FITC labeled *S. pneumoniae* for 4 h and in vivo phagocytosis by AM (**I–J**) and neutrophils (**K–L**) was determined. **J** and **L** show representative FACS plots of data in **I** and **K**. (**M**) WT and *Trem-2* ^−/−^ mice (n = 6 mice per genotype) were intranasally infected with 10^5^ CFU *S. pneumoniae* and bacterial CFUs were enumerated 24 h post infection in the lung and BALF. All data represent mean ± SEM versus WT unless otherwise indicated. Data in (**A–C, F and H**) are representative of three independent experiments and all other data are representative of two independent experiments. * p<0.05, ** p<0.005, **** p<0.0001.

Considering that the elimination of pathogens is the crucial step in host defense during pneumonia, we then more closely examined the anti-bacterial properties of macrophages and the role of TREM-2 herein. In agreement with published reports that indicated TREM-2 to be a phagocytic receptor for *E. coli* [21] we first identified that *Trem-2* ^−/−^ BMDM exhibited a decreased uptake of *S. pneumoniae* compared to WT cells (**Fig. 3A**). Although we anticipated that *Trem-2* ^−/−^ AM would behave similarly, this postulate proved to be incorrect as *Trem-2* ^−/−^ AM surprisingly exhibited an enhanced uptake of *S. pneumoniae* compared to WT AM (**Fig. 3B**). We could rule out differences in phagocytosis between the cell-types due to the kind of bacterium used, since uptake of *E. coli* was equally enhanced in *Trem-2* ^−/−^ AM compared to WT AM (**Fig. 3C**). We further confirmed the enhanced uptake of *S. pneumoniae* in *Trem-2* ^−/−^ AM using confocal microscopy (**Fig. 3D** **and Fig. S1**) and corroborated previously published observations showing that *Trem-2* ^−/−^ BMDM exhibit decreased uptake of *E. coli* compared to WT BMDM [21] (**Fig. S2**). Increased phagocytosis of *S. pneumoniae* by TREM-2 deficient AM was also found following opsonisation of bacteria with either pneumococcal serotype 3 capsular antibodies or 10% WT serum, indicating that TREM-2 can inhibit both FcR-dependent and independent phagocytosis of *S. pneumoniae* (**Fig. 3E and F**). Uptake of FITC labeled BSA was unchanged between the genotypes of AM, while in the same experiment uptake of *S. pneumoniae* was significantly higher, thus illustrating that TREM-2 affects phagocytosis of bacteria but not endocytosis of BSA (**Fig. 3G**). Further, to extend these studies to other serotypes of *S. pneumoniae*, we tested uptake of serotype 19A *S. pneumoniae*, a common serotype that causes invasive pneumococcal disease in children [38]. By doing so, we could observe enhanced uptake of 19A *S. pneumoniae* in *Trem-2* ^−/−^ AM compared to their WT counterparts (**Fig. 3H**).

We next set out to investigate if the enhanced phagocytosis of *S. pneumoniae* by TREM-2 deficient AM would also be observed in vivo. To this end, we inoculated WT and *Trem-2* ^−/−^ mice with FITC-labeled *S. pneumoniae* and assessed the uptake of *S. pneumoniae* by phagocytes in vivo using flow cytometry. By doing so, we could verify that *Trem-2* ^−/−^ F4/80^+^ CD11c^+^ AM did indeed display augmented bacterial phagocytosis in vivo compared to their WT counterparts (**Fig. 3I and J**). Considering the importance of infiltrating neutrophils in phagocytosing bacteria during pneumonia, we then examined the potential contribution of neutrophils to bacterial clearance in vivo. Following intranasal infection with FITC labeled *S. pneumoniae*, we found a tendency for decreased phagocytosis by Ly6G^+^CD11b^+^ lung neutrophils from *Trem-2* ^−/−^ mice compared to their WT counterparts, suggesting that the modest increase in neutrophil numbers early during infection (**Fig. 1G**) was not responsible for the improved bacterial clearance of *Trem-2* ^−/−^ mice (**Fig. 3K and L**). We finally asked if the enhanced phagocytosis by *Trem-2^−/−^* AM would result in an improved bacterial clearance during pneumococcal pneumonia in vivo. We infected mice with *S. pneumoniae* for 24 h and recovered significantly fewer bacteria from lungs and bronchoalveolar lavage fluid (BALF) from *Trem-2^−/−^* mice (**Fig. 3M**).

These data were surprising and important because they challenged the general view of TREM-2 acting as a phagocytic receptor and negative regulator of inflammation but suggested that TREM-2 can modulate phagocytosis and inflammation in a very cell-type specific manner. With uptake of bacteria being higher in resident AM from TREM-2 deficient mice, this macrophage type obviously displays opposite effects from BMDM.

### Increased C1q production via PPAR-δ in TREM-2-deficient alveolar macrophages

**Figure 4:**
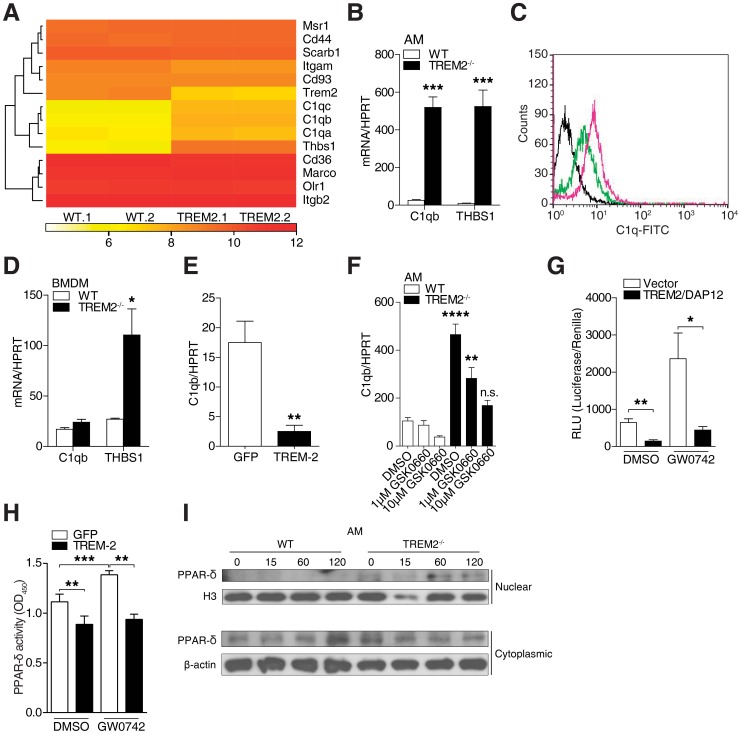
Elevated C1q production via PPAR-δ in TREM-2 deficient AM. (**A**) Heat map from microarray data depicting baseline expression of selected genes in WT and *Trem-2* ^−/−^ AMs. (**B**) Verification of enhanced basal expression of the opsonins *C1qb* and *Thbs1* in AM using RT-PCR (n = 4 per genotype). (**C**) Basal expression of *C1qb* in WT versus *Trem-2* ^−/−^ AMs as determined by intracellular FACS. Green line depicts WT macrophages, pink *Trem-2* ^−/−^ macrophages and black represents isotype control antibody. (**D**) *C1qb* and *Thbs1* basal expression was determined in WT and *Trem-2* ^−/−^ BMDM (n = 4 per genotype) using RT-PCR. (**E**) *C1qb* expression was quantified in RAW264.7 cells over-expressing TREM-2 or GFP control (n = 4 per condition). (**F**) WT and *Trem-2* ^−/−^ AM (n = 4 per genotype/condition) were pre-treated for 24 h with the indicated doses of the PPAR-δ inhibitor GSK0660 or DMSO control after which RT-PCR of *C1qb* was performed. (**G**) HEK cells were transfected with a PPRE reporter plasmid together with TREM-2 and DAP-12 or a vector control, stimulated with 1 µM of the PPAR-δ activator GW0742 or DMSO 24 h post transfection, and luciferase activity was assayed 48 h post transfection (n = 4 per condition). (**H**) RAW264.7 cells over-expressing TREM-2 or GFP control (n = 4 per condition), were treated with 1 µM of the PPAR-δ activator GW0742 or DMSO control for 24 h, nuclear extracts were prepared and PPAR-δ activity levels were monitored as described in the methods. (**I**) WT and *Trem-2* ^−/−^ AM were treated with 1 µM of the PPAR-δ activator GW0742 or DMSO control for the indicated time points, nuclear and cytoplasmic extracts were prepared and blotted for PPAR-δ. All data are representative of two independent experiments except for data in (**E**), which is representative of three independent experiments, and represent mean ± SEM versus WT (**B and D**), GFP control cells (**E**), DMSO (**F**) or vector (**G**). * p<0.05, ** p<0.005, *** p<0.001, **** p<0.0001.

Given the importance of lung macrophages in pneumococcal clearance [29], [39], we next focused on determining the molecular targets by which TREM-2 deficiency in AM was able to influence pneumococcal uptake and we conducted a genome wide transcriptome analysis of both genotypes of AM. While we discovered no difference in the expression levels of important phagocytic receptors such as the scavenger receptors *Cd36*, *Marco*, *Sra-1* and *Lox-1* and the complement receptors *Cr1 (Cd35)* and *Cr3 (itgb2/itgam; Cd11b/Cd18)* (**Fig. 4A** and **Fig. S3**), we importantly found that *Trem-2* ^−/−^ AM expressed higher baseline levels of opsonins such as *C1qa*, *C1qb*, *C1qc* and *Thbs1* (encoding thrombospodin; **Fig. 4A**). We confirmed higher basal *C1qb* and *Thbs1* levels in TREM-2 deficient AMs by RT-PCR (**Fig. 4B**) and verified enhanced intracellular C1q protein levels in *Trem-2* ^−/−^ AM by flow cytometry (**Fig. 4C**). In search for specific factors that would explain differences in TREM-2 mediated responses between AM and BMDM, we found that *C1qb* levels were exclusively increased in *Trem2^−/−^* AM, whereas *Thbs1* levels were elevated in both TREM-2 deficient AM and BMDM (**Fig. 4D**). This led us to hypothesize that altered C1q expression could account for differential phagocytic effects between AM and BMDM.

As microarray data suggested that TREM-2 had the ability to influence basal C1q production in AM, we decided to test this idea and to determine *C1qb* and *C1qc* transcript levels in RAW264.7 macrophages over-expressing TREM-2. Over-expression of TREM-2 was able to lower basal *C1qb* and *C1qc* transcript levels, proving that TREM-2 had the ability to regulate C1q transcription (**Fig. 4E** **and Fig. S4**). The nuclear receptor peroxisome proliferator-activated receptor-δ (PPAR-δ) has previously been shown to regulate C1q and the C1q promoter contains binding sites for this transcription factor [40]. To understand the potential role of PPAR-δ in our cell system, we treated *Trem-2* ^−/−^ AM with the PPAR-δ inhibitor GSK0660 [41] and could revert *C1qb* transcript levels to those of WT AM in a dose dependent manner (**Fig. 4F**). We observed similar results using another PPAR-δ inhibitor, arguing against inhibitor-specific effects in down regulating C1q expression (**Fig. S5**). Specificity of these compounds for PPAR-δ and not other PPARs such as PPAR-γ was evaluated, since activation of PPAR-δ with the PPAR-δ activator GW0742 [40] increased transcript levels of *C1qb* and *C1qc* in WT AM in a dose dependent manner and co-incubation with PPAR-δ inhibitors abrogated this increase (**Fig. S5**). These data confirmed that PPAR-δ activity determines C1q transcription in AM. There was no striking difference in transcript and total protein levels of PPAR-δ between WT and *Trem-2^−/−^* AM, suggesting that the ability of TREM-2 to regulate C1q via PPAR-δ was not due to enhanced PPAR-δ levels (**Fig. S6**). To demonstrate that TREM-2 itself downregulates PPAR-δ activity, we made use of TREM-2 over-expressing HEK cells and quantified PPAR-δ activation using a reporter system. Over-expression of TREM-2 was able to lower both basal and ligand-induced PPAR-δ reporter activity (**Fig. 4G**). To rule out differences between HEK cells and macrophages, and to further confirm the reporter gene experiments, we next stimulated TREM-2 or GFP control over-expressing RAW264.7 macrophages with GW0742 or DMSO control and monitored nuclear PPAR-δ levels. These data corroborated the experiments in Fig. 4G and demonstrated that TREM-2 could suppress PPAR-δ activity in RAW264.7 macrophages (**Fig. 4H**). To understand the specific effect of TREM-2 on PPAR-δ activation in AM, we next monitored nuclear and cytoplasmic levels of PPAR-δ in WT and *Trem-2* ^−/−^ AM following GW0742 treatment. *Trem-2* ^−/−^ AM exhibited an early translocation of ligand-induced PPAR-δ from the cytoplasm to the nucleus, compared to their WT counterparts (**Fig. 4I**).

Altogether, these data demonstrate that TREM-2 can suppress basal levels of C1q and suggest that the elevated C1q production observed in *Trem-2* ^−/−^ AM occurs via effects of TREM-2 on the nuclear receptor PPAR-δ, a known activator of C1q transcription [40].

### Elevated *S. pneumoniae* phagocytosis and bacterial clearance in TREM-2 deficient AM and mice is dependent on C1q

C1q is unique among the complement factors, as it is exclusively produced by macrophages and not hepatocytes [42], [43], [44], [45], [46]. While C1q is well-known for its role in initiating the classical complement pathway, it also exerts complement-independent functions such as clearance of immune-complexes or apoptotic cells [47], [48], [49]. The importance of complement in general, including C1q, in protecting against pneumococcal infections is well established [30], [50] and has been based on the importance of C3b-mediated opsonization of bacteria. Since we observed that TREM-2 deficient AM produced more C1q (**Fig. 4A, B, C and F**) and that *Trem-2* ^−/−^ mice showed a modestly elevated neutrophil influx early during pneumococcal pneumonia (**Fig. 1G**), we speculated that classical complement activation could be increased in the pulmonary compartment of *Trem-2* ^−/−^ mice following *S. pneumoniae* infection and monitored levels of C3a and C5a, anaphylatoxins, which promote neutrophil influx [51]. However, we did not observe any differences in C3a and C5a in the BALF of *Trem-2* ^−/−^ mice both 6 h and 24 h post *S. pneumoniae* infection, suggesting that classical complement activation was not generally elevated in the pulmonary compartment of *Trem-2* ^−/−^ mice following *S. pneumoniae* infection (**Fig. S7**).

**Figure 5:**
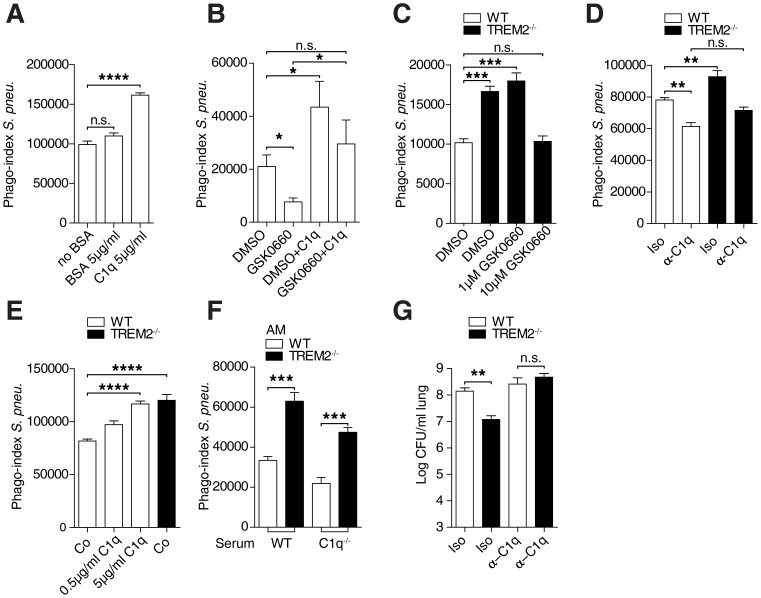
Enhanced phagocytosis by *Trem-2* ^−/−^ AM depends on C1q. (**A**) WT AM were adhered to C1q, control BSA or uncoated plates for 3 h prior to incubation with FITC labeled *S. pneumoniae* (MOI 100) and phagocytosis was assessed using FACS 1 h later (n = 3–4 per condition). (**B**) WT AM (n = 6–7 per condition) were pre-treated for 24 h with 10 µM PPAR-δ inhibitor GSK0660 or DMSO control after which, the cells were adhered to C1q or control plates for 3 h and phagocytosis of FITC labeled *S. pneumoniae* (MOI 100) was assessed 1 h later using FACS. (**C**) WT and *Trem-2* ^−/−^ AM (n = 4 per genotype/condition) were pre-treated for 24 h with the indicated doses of the PPAR-δ inhibitor GSK0660 or DMSO control after which phagocytosis of FITC labeled *S. pneumoniae* (MOI 100) was assessed 1 h later using FACS. (**D**) WT and *Trem-2* ^−/−^ AM were pre-treated for 1 h with 10 µg/ml C1q blocking antibody or isotype control and phagocytosis of FITC labeled *S. pneumoniae* (MOI 100) was assessed 1 h later using FACS (n = 4 per genotype/condition). (**E**) WT and *Trem-2* ^−/−^ AM (n = 4–7 per condition) were adhered for 3 h to plates coated with the indicated conditions prior to incubation with FITC labeled *S. pneumoniae* (MOI 100) and phagocytosis was assessed using FACS. (**F**) WT and *Trem-2* ^−/−^ AM (n = 4 per genotype) were incubated with FITC labeled *S. pneumoniae* (MOI 100) that were pre-opsonised with 10% WT or C1qa^−/−^ serum for 30 min after which phagocytosis was assessed 1 h later using FACS (**G**) WT and *Trem-2* ^−/−^ mice (n = 17 per condition) were intranasally treated with 125 µg C1q blocking antibody or isotype control prior to infection with 6×10^4^ CFU *S. pneumoniae*. 48 h post infection lung bacterial CFUs were enumerated. Data represent mean ± SEM; ** p<0.005, *** p<0.001, **** p<0.0001. Data in (**A, C–F**) are representative of two independent experiments, (**B and G**) are pooled data from two independent experiments.

We therefore studied the potential direct contribution of C1q to bacterial phagocytosis, and pre-incubated WT AM with C1q or control BSA and quantified the uptake of *S. pneumoniae*. C1q significantly increased bacterial phagocytosis by AM (**Fig. 5A**), thus confirming a direct role for C1q in phagocytosis, independent of complement activation. Further, these data are consistent with data, showing that C1q can bind to *S. pneumoniae*, independent of IgG and increase internalization [52]. As our data indicated that PPAR-δ activation enhanced C1q levels in WT AM (**Fig. S5**) and that PPAR-δ inhibition could lower C1q levels (**Fig. 4F**), we next decided to examine the role of PPAR-δ mediated C1q production in the context of *S. pneumoniae* phagocytosis within WT AMs. AMs, where PPAR-δ had been inhibited exhibited lower *S. pneumoniae* uptake compared to DMSO control cells. Importantly, the attenuated *S. pneumoniae* phagocytosis upon PPAR-δ inhibition was increased to levels of DMSO control cells upon addition of C1q (**Fig. 5B**). These data strongly suggest that in AM the principal target for PPAR-δ is C1q for the regulation of *S. pneumoniae* phagocytosis. Given that PPAR-δ inhibitors can lower basal C1q production in *Trem-2* ^−/−^ AM (**Fig. 4F** **and Fig. S5**), we next postulated that inhibiting C1q using PPAR-δ inhibitors would revert the enhanced phagocytosis by *Trem-2^−/−^* AM to WT levels. Significantly, at the dose of inhibitor where *C1qb* transcript levels in TREM-2 deficient AM were lowered to that of WT AM (i.e. 10 µM GSK0660 (**Fig. 4F**)), phagocytosis was no longer different between WT and *Trem-2* ^−/−^ AM (**Fig. 5C**). These data linked elevated C1qb levels, produced in a PPAR-δ dependent manner, with enhanced phagocytosis. To confirm the dependence of enhanced phagocytosis by *Trem-2* ^−/−^ AM on higher C1q levels, we blocked C1q using a blocking antibody and discovered that C1q blockage abolished any differences in phagocytosis between WT and *Trem-2^−/−^* AM (**Fig. 5D**). To then test if exogenous administration of C1q would enhance phagocytosis of WT AMs to *Trem-2* ^−/−^ levels, we adhered WT AM to C1q coated plates and quantified uptake of *S. pneumoniae* compared to *Trem-2* ^−/−^ AM. Consistent with results in Fig. 5A, C1q enhanced phagocytosis of *S. pneumoniae* by WT AM in a dose dependent manner to reach *Trem-2* ^−/−^ levels (**Fig. 5E**). Thus, using two independent approaches of inhibiting C1q in *Trem-2* ^−/−^ AM, as well as exogenously supplementing C1q in WT AM, we demonstrated that the enhanced phagocytosis of *S. pneumoniae* by *Trem-2* ^−/−^ AM depended on macrophage derived C1q.

Bone marrow transplantation experiments indicate that transplantation of WT bone marrow into *C1qa^−/−^* mice restores levels of serum C1q to WT, clearly demonstrating that C1q is produced by myeloid cells [46]. To further ratify the importance of macrophage derived C1q versus serum C1q, we next opsonised *S. pneumoniae* with serum from WT or *C1qa^−/−^* mice and examined phagocytosis. Our hypothesis was that the difference in phagocytosis between WT and *Trem-2^−/−^* AM would still be visible in the presence of C1q-deficient serum, although overall phagocytosis levels would be reduced in both genotypes. Indeed, this was the case, strongly suggesting that AM are the source of C1q and that elevated C1q levels produced by *Trem-2^−/−^* AM mediate the enhanced phagocytosis of *S. pneumoniae* (**Fig. 5F**).

To finally study the importance of elevated C1q production by *Trem-2* ^−/−^ AM in vivo, we blocked C1q in lungs of mice before infection with *S. pneumoniae* and quantified bacterial clearance. Indeed, blocking pulmonary C1q could reverse the difference in bacterial counts between WT and *Trem-2* ^−/−^ animals (**Fig. 5G**). We conclude that the improved bacterial clearance we observed in TREM-2 deficient animals was intimately linked to enhanced C1q production by TREM-2 deficient AM.

### TREM-2 is deleterious during pneumococcal pneumonia

**Figure 6:**
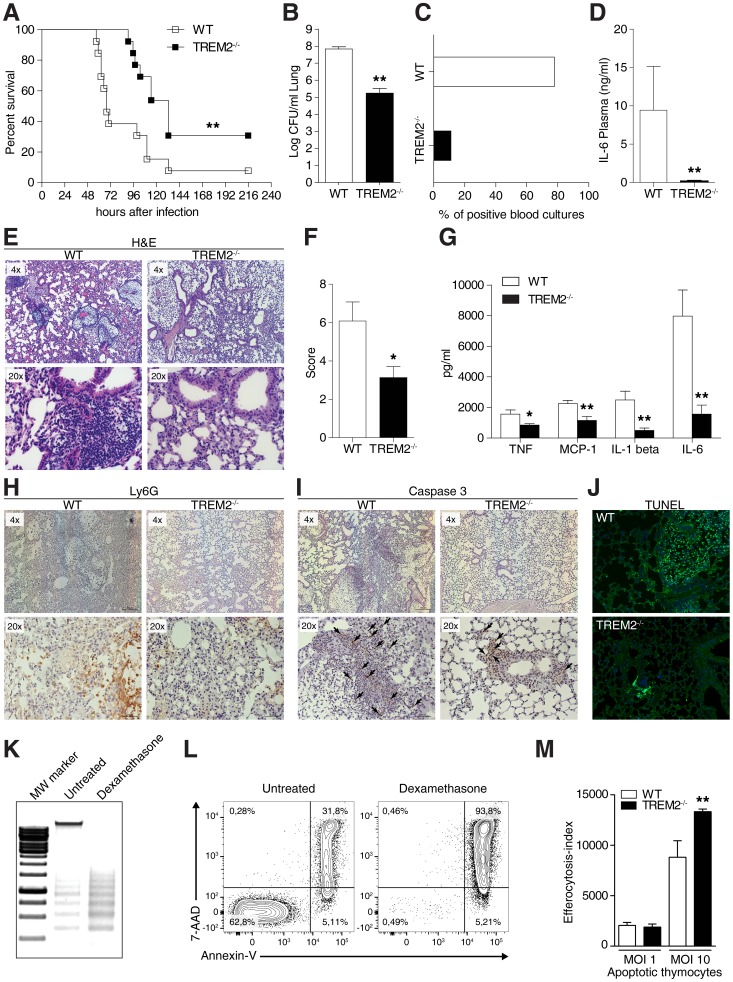
TREM-2 deficiency improves outcome during pneumococcal pneumonia. (**A**) WT and *Trem-2* ^−/−^ mice were intranasally infected with 10^5^ CFU *S. pneumoniae* and survival was monitored for 10 days (n = 13/genotype). (**B–G**) WT and *Trem-2* ^−/−^ mice (n = 9 mice per genotype) were intranasally infected with 1×10^5^ CFU *S. pneumoniae* and (**B**) lung bacterial CFUs were enumerated 48 h post infection. (**C**) Blood cultures were monitored for *S. pneumoniae* (**D**), IL-6 levels were evaluated in the plasma using ELISA. (**E**) Representative H/E staining of lungs 48 h post infection. (**F**) Lung inflammation score, as described in the Methods section. (**G**) Levels of lung cytokines were evaluated using ELISA. (**H–J**) Representative Ly6G (**H**), active caspase 3 (**I**) and TUNEL (**J**) staining of lungs 48 h post infection. Magnification depicted for TUNEL stains is 20× and arrows indicate caspase 3 positive cells. (**K–L**) Thymocytes (n = 4) were treated with 1 µM dexamethasone and apoptosis was evaluated using DNA laddering (**K**) or Annexin-V/7-AAD positivity (**L**). (**M**) WT and *Trem-2* ^−/−^ AM (n = 8 per genotype and condition), were fed CFSE labeled apoptotic cells and efferocytosis was assessed 1 h later using FACS. Data in **B**, **D**, **F**, **G** and **M** are presented as mean ± SEM, WT versus TREM-2^−/−^. Data in **A–G** are representative of two independent experiments. Data in **M** is pooled data from 2 independent experiments; * p = <0.05, ** p = 0.005.

The enhanced bacterial phagocytosis of *S. pneumoniae* in *Trem-2* ^−/−^ mice finally led us to monitor the survival of WT and *Trem-2* ^−/−^ mice during pneumonia. Ninety five hours post infection, before the first *Trem-2* ^−/−^ mouse succumbed, 60% of WT mice were dead, demonstrating conclusively that WT mice displayed enhanced mortality compared to their TREM-2 deficient counterparts during pneumococcal pneumonia (**Fig. 6A**). To determine the reasons for this effect, we evaluated pulmonary bacterial loads and bacteremia shortly before the first mouse succumbed. TREM-2 deficient mice exhibited a thousand fold decrease in *S. pneumoniae* burden in lungs 48 h post infection (**Fig. 6B**). These data corroborated our earlier time points of infection (**Fig. 3M**). However, although, we had observed that *Trem-2* ^−/−^ mice reproducibly exhibit enhanced bacterial clearance compared to WT mice (**Fig. 3M** **,** **5G** **and** **6B**), there were some differences in the degree of bacterial clearance. Natural variation in animal experiments or differences in experimental setup could explain this. Strikingly, while seven out of nine WT mice exhibited bacteremia, *S. pneumoniae* could only be detected in the blood of one *Trem-2* ^−/−^ mouse (**Fig. 6C**). Elevated bacteremia in WT mice resulted in enhanced systemic inflammation as determined by plasma IL-6 measurements, compared to TREM-2 deficient mice (**Fig. 6D**).

We next evaluated lung pathology and inflammation. Lung histology revealed that the levels of interstitial inflammation, pleuritis and edema formation were greatly decreased in mice deficient for TREM-2 compared to WT mice (**Fig. 6E and 6F**). In agreement with this, we detected diminished pulmonary cytokine levels such as TNF-α, IL-1β, MCP-1 and IL-6 (**Fig. 6G**).

Attenuated pulmonary inflammation is not only associated with decreased bacterial burden within the lungs but can also be attributed to improved clearance of apoptotic neutrophils by AM, which promotes the resolution of inflammation [53], [54], [55], [56]. To examine the possibility that the higher inflammation in WT mice might be associated with more apoptotic cells, we monitored pulmonary neutrophil infiltration and active caspase 3 levels in WT and *Trem-2^−/−^* mice using Ly6G and active caspase 3 staining. We could observe that *Trem-2^−/−^* mice displayed attenuated neutrophil and active caspase 3 levels, particularly in the interstitial space, 48 h post *S. pneumoniae* infection (**Fig. 6H, 6I**). Elevated pulmonary cell apoptosis of WT mice late during pneumococcal pneumonia was confirmed using TUNEL staining (**Fig. 6J**). Specificity for all stainings was verified as no positive signal was detected in the respective isotype controls (**Fig. S8**). We next tested the hypothesis that aside from improved *S. pneumoniae* phagocytosis (**Fig. 3** **and** **5**), *Trem-2^−/−^* AM may exhibit elevated apoptotic cell uptake, also known as efferocytosis. To model this we measured the uptake of CFSE labeled apoptotic thymocytes, a well-established and widely used method for determining efferocytosis. Thymocyte apoptosis was confirmed using both DNA laddering and Annexin V/7-AAD positivity (**Fig. 6K and L**). While there was no difference in the clearance of CFSE labeled apoptotic bodies at low doses between the genotypes of AM, importantly, at a MOI of 10, *Trem-2^−/−^* AM exhibited significantly elevated efferocytosis compared to WT AM (**Fig. 6M**).

We conclude that TREM-2 deficiency improved bacterial and apoptotic cell clearance, lung pathology and prevented systemic inflammation during pneumococcal pneumonia, all of which ultimately led to improved survival.

## Discussion

In this study we examined the effects of TREM-2 on bacterial phagocytosis and pulmonary inflammation within the context of bacterial pneumonia. We unexpectedly discovered a cell-type specific role for TREM-2, as TREM-2 suppresses bacterial phagocytosis via repression of C1q in AM. These findings demonstrate a previously unknown link between ITAM associated receptor expression and opsonin production in resident AM and explains the detrimental function of TREM-2 during pneumococcal pneumonia.

C1q is a member of the defense collagen family that is important for initiating the classical complement pathway and thereby crucial for host defense against pneumococci [30], [50], [57]. C1q consists of 18 polypeptide chains that associate together in a “bouquet of tulips” like configuration, with each C1q chain containing a C-terminal globular region that recognizes PAMPs, and a N-terminal collagenous region that associates with phagocytic receptors on macrophages to enhance bacterial phagocytosis [45], [51]. Within the pulmonary compartment, C1q is produced locally by AM [45], [46], i.e. by those cells that provide the first phagocytic line of defense against *S. pneumoniae* [29], [39]. C1q has been shown to act as a molecular bridge between *S. pneumoniae* and host cells, independently of IgG and serotype, facilitating increased adherence and bacterial uptake [52]. Our data reveal a previously unknown link between C1q and TREM-2 and suggest that TREM-2 suppresses C1q production by AM via a mechanism that involves PPAR-δ associated pathways (**Fig. 4**). The consequence of this suppressive effect by TREM-2 in AM is important as it explains the detrimental impact of TREM-2 during pneumococcal pneumonia. Our findings that blocking the locally enhanced production of pulmonary C1q was sufficient to reverse the improved bacterial clearance by *Trem-2^−/−^* AM in vivo and in vitro support this argument (**Fig. 5**).

Beside AM, neutrophils are considered important in phagocytosing bacteria upon lung infections and since we observed a modest increase in neutrophil influx early during pneumococcal pneumonia in *Trem-2* ^−/−^ mice, these cells could contribute to the enhanced bacterial clearance in these mice. Although we cannot exclusively rule out this possibility, we importantly discovered that neutrophils from TREM-2 deficient mice exhibit a tendency towards lower uptake of *S. pneumoniae* in vivo, while *Trem-2* ^−/−^ AM exhibit increased phagocytosis in vitro and in vivo (**Fig. 3**). Underscoring the importance of AM in bacterial clearance during pneumonia over neutrophils, previous studies by our group show that selective changes in KC secretion and neutrophil influx occurring in a TLR2 dependent manner are not sufficient to induce altered bacterial clearance or differences in outcome during pneumococcal pneumonia in vivo [58].

But how does TREM-2 influence C1q production? PPARs are nuclear receptors that are ligand inducible transcription factors and activate target genes through binding to PPAR-response elements (PPREs) as heterodimers with the retinoid X receptor family [59]. PPARs shuttle between the cytoplasm and nucleus in response to ligand activation [60]. Three lines of evidence link PPAR-δ to the enhanced C1q production in AM: 1) PPAR-δ activation enhances production of C1q by AM; 2) PPAR-δ inhibition lowers levels of C1q in *Trem-2* ^−/−^ AM to WT AM; 3) Over-expression of TREM-2 lowers levels of C1q and PPAR-δ activation. Interestingly the early ligand induced activation kinetics and nuclear shuttling of PPAR-δ are elevated in *Trem-2* ^−/−^ AM, while PPAR-δ appears to be primarily localized in the cytoplasm of WT AM (**Fig. 4I**). These data suggest that the manner by which TREM-2 influences C1q transcription is mediated by effects on PPAR-δ activation, possibly via interference with PPAR-δ ligand binding, as already suggested by our PPRE reporter experiments and activity assays (**Fig. 4G and H**). Regardless of the exact mechanism whereby TREM-2 inhibits PPAR-δ activity, our data suggests that C1q is the principal target for PPAR-δ in the regulation of *S. pneumoniae* phagocytosis within AM (**Fig. 5B–C**).

Our observations of enhanced phagocytosis in TREM-2 deficient AM are important as they raise awareness of conceptually labeling “TREM-2 as a phagocytic receptor for bacteria.” We do not dispute previous studies showing that TREM-2 deficiency in BMDM leads to lower bacterial phagocytosis [21]. In fact, we were able to reproduce these findings but believe that the effects of TREM-2 on phagocytosis are cell-type specific. While AM and BMDM are related cell types, they present substantial differences with the former cell type being a resident macrophage isolated using lavage and the latter isolated from bone marrow and in vitro differentiated with M-CSF. Although differential C1q expression upon TREM-2 deficiency may account for the phagocytic differences between the cell types, it is highly likely that in BMDM, additional factors play a role.

The cell-type specific effects of TREM-2 on phagocytosis are reminiscent of its effects on antigen presentation and osteoclastogenesis, which clearly differ between different cell types. TREM-2 stimulation of immature dendritic cells induces expression of MHC class II and co-stimulatory molecules that are required for antigen presentation [61], but this effect is not observed in microglia [11]. TREM-2 deficiency in RAW 264.7 macrophages and human monocytes leads to a reduced capacity to generate osteoclast precursors but bone marrow cells from *Trem-2* ^−/−^ mice exhibit accelerated osteoclastogenesis [12], [32], [62]. Furthermore, although TREM-2 has been shown to be a phagocytic receptor for apoptotic neurons [11], uptake of microspheres is unchanged following knockdown of TREM-2, indicating TREM-2 is not essential for all types of phagocytosis [63].

Interestingly, our data show that AM expressed TREM-2 not only suppresses bacterial phagocytosis but also efferocytosis (**Fig. 6M**). These data are in contrast to previous studies suggesting that TREM-2 promotes the uptake of apoptotic neurons by microglia [11], [63]. C1q is important for the uptake of apoptotic cells [47], [48], [49]. Indeed, as C1qa^−/−^ mice age they develop multiple apoptotic bodies and autoimmunity compared to WT controls [47]. Within the lung, improved clearance of apoptotic neutrophils by AM promotes the resolution of inflammation [53], [54], [55], [56]. Our data raise the intriguing possibility, that AM expressed TREM-2 might influence efferocytosis through C1q. This hypothesis is consistent with our observations of TREM-2 specifically modulating bacterial phagocytosis and efferocytosis but not the uptake of BSA by AM. These findings make perfect sense in the context of TREM-2 modulating these processes via C1q, as C1q acts as a bacterial opsonin and is also critical for the uptake of apoptotic cells.

The ability of TREM-2 to regulate cellular responses in a cell-type specific manner is not limited to phagocytosis and efferocytosis. In this study we show that TREM-2 also modulates *S. pneumoniae* and TLR2 induced inflammation in a cell-type specific manner, with TREM-2 deficient AMs displaying less inflammation in response to either stimulus. Importantly, these observations are consistent with very recent studies by Correale *et al.*, who demonstrated that dendritic cells from *Trem-2* ^−/−^ mice display attenuated inflammation in response to TLR ligands [64]. Further, we find in vivo that, other than KC, no other inflammatory mediator tested was higher in *Trem-2* ^−/−^ mice following early pneumococcal infection compared to their WT counterparts. In this regard it is important to note that recent observations indicate that the anti-inflammatory activity of TREM-2 in vivo may differ depending on the disease context. For example, *Trem-2* ^−/−^ mice exhibit attenuated inflammation following DSS induced colitis and stroke, rather than augmented inflammation as expected [64], [65]. In summary, our study, together with others present in the literature, provides credence for the claim that TREM-2 impacts cellular responses in a cell-type, stimulus- and disease context-specific manner. Since we found TREM-2 to suppress C1q secretion, it is interesting that C1q has been shown to dampen TLR mediated cytokine synthesis, although the exact mechanism behind this is unknown [66], [67]. It is tempting to speculate that the suppressive effects of TREM-2 on C1q production in AM not only modulate bacterial phagocytosis but also dampen TLR mediated inflammation.

This is the first report demonstrating a function for TREM-2 in the pulmonary compartment. Importantly, we clearly show that the ability of TREM-2 to confer bacterial phagocytosis is cell-type specific and that TREM-2 modulates C1q production by AM. It is tempting to speculate that targeting the TREM-2 pathway could be used as a novel strategy for modulating C1q production and pulmonary innate immune responses, which might be of relevance to other respiratory tract infections and possibly autoimmune diseases.

## Materials and Methods

### ELISA and reagents

ELISA kits for mouse TNF, MCP-1, IL-1β, IL-6, KC and C5a were from R & D Systems and the mouse C3a ELISA was from Uscn Life Science Inc. All ELISAs were performed according to the manufacturer's instructions. GSK0660 was from Tocris Biochemicals. GW0742 and GSK3787 and recombinant C1q were purchased from Sigma. *S. pneum*oniae LTA was a kind gift from Sonja von Aulock (University of Konstanz, Germany). C1q antibodies used for FACS and blocking experiments were clones 7H-8 (in vitro and in vivo) and JL-1 (in vitro) respectively, both purchased from Hycult Biotech as was the MARCO (clone ED-31) antibody. Isotype control antibody IgG2bκ (559530) used for C1q blocking experiments was from BD Bioscience. CD36 (clone 63) antibody was from Millipore. CD45-V500 (clone 30-F11) was from BD Bioscience. Ly6G-PE (clone 1A8), CD11c-APC (clone N418), F4/80 (clone BM8) antibodies were from Biolegend. CD11b-Alexa Fluor 700 (clone M1/70) was from eBioscience. TREM-2 (BAF1729), PPAR-δ ab8937) and β-actin (clone AC15) antibodies for western blotting were from R/D systems, AbCaM and Sigma respectively. Recombinant PPAR-δ was supplied in the PPAR-δ activity assay (Abcam ab133106). Ly6G (clone 1A8) and active caspase 3 (Asp175 – clone 5A1E) antibodies used in IHC were from BD Bioscience and Cell Signaling respectively. As secondary reagents we used PE conjugated rat anti-mouse (eBioscience), FITC conjugated goat anti-rat F(ab')_2_ (Jackson Immunoresearch), anti-rabbit HRP (Cell Signaling), streptavidin HRP (R/D systems), anti-mouse HRP (BioRad), biotinylated anti-rat IgG (Vector Laboratories) and biotinylated, swine anti-rabbit IgG (Dako) .

### Animals

*Trem-2* ^−/−^ mice were generated as previously described [10] and the TREM-2 mutation was backcrossed onto a >98% B6 C57BL/6 background facilitated by genome-wide SSLP typing at 10 cM intervals (done by the Speed Congenics Facility of the Rheumatic Diseases Core Center). Wild type mice were purchased from Charles River and all mice were bred at the Medical University of Vienna Animal facility under pathogen free conditions. Age (8–10 week) and sex matched mice were used in all experiments.

### Ethics statement

All animal experiments were discussed and approved through the Animal Care and Use Committee of the Medical University of Vienna and the Austrian Ministry of Sciences and were carried out in strict accordance with Austrian law (Tierversuchsgesetz; BMWF-66.009/0321-II/10b/2008).

### Bacteria

*S. pneumoniae* serotype 3 was obtained from American Type Culture Collection (ATCC 6303). Serotype 19A *S. pneumoniae* was a clinical isolate from a patient suffering from severe invasive pneumococcal disease and confirmation of the serotype was provided by antibodies specific to the capsule (Statens Serum Institute). Both strains were grown for 6 h to mid-logarithmic phase at 37°C in Todd-Hewitt broth (Difco), harvested by centrifugation at 4000 rpm for 15 min at 4°C, and washed twice in sterile saline. *S. pneumoniae* serotype 3, was used for all in vivo experiments and most in-vitro experiments (except Fig. 3H). Bacteria were diluted in sterile saline to obtain an estimated concentration of 10^5^ CFU per 50 µl for intranasal inoculation of mice. The true concentration was determined by growing serial 10-fold dilutions on sheep blood agar plates overnight.

### Mouse model of pneumococcal pneumonia

Pneumonia was induced by intranasal administration of a bacterial suspension containing 10^5^ CFU *S. pneumoniae* (ATCC 6303) as described earlier [19], [58], [68]. Six, 24, or 48 h after infection, mice were anesthetized with ketamine (Pfizer) and sacrificed. Blood was collected in EDTA-containing tubes and plated on blood agar plates to determine bacterial counts, plasma was stored at −20°C. Whole lungs were homogenized at 4°C in 4 volumes of sterile saline using a tissue homogenizer (Biospec Products), after which serial 10-fold dilutions in sterile saline were plated on blood agar plates, left at 37°C and CFU were counted 16 h later. Remaining lung homogenates were incubated for 30 min in lysis buffer (containing 300 mM NaCl, 30 mM Tris, 2 mM MgCl_2_, 2 mM CaCl_2_, 1% Triton X-100, and pepstatin A, leupeptin, and aprotinin (all 20 ng/ml; pH 7.4; Sigma-Aldrich)) at 4°C, centrifuged at 1500 *g* at 4°C, and supernatants were stored at −20°C until cytokine measurements were performed. Lungs for histology were harvested 48 h post infection, fixed in 10% formalin, and embedded in paraffin. Four-µm sections were stained with H&E and analyzed by a pathologist blinded for groups, who scored lung inflammation and damage as previously described [19], [58].

In separate experiments, bronchoalveolar lavage (BAL) was performed 6 h and 24 h after infection by exposing the trachea of mice through a midline incision, canulating it with a sterile 18-gauge venflon (BD Biosciences) and instilling two 500 µl aliquots of sterile saline. Approximately, 0.9 ml was retrieved per mouse. Total cell numbers were counted using a hemocytometer (Türck chamber); differential cell counts were done on cytospin preparations stained with Giemsa. BALF supernatant was stored at −20°C for cytokine measurements. In some experiments, to assess survival, mice were intranasally infected with *S. pneumoniae* and survival was monitored regularly for 10 days. In experiments involving C1q blocking, 125 µg of C1q blocking antibody (kindly provided by Admar Verschoor, Technical University of Munich) or isotype control were intranasally instilled prior *to S. pneumoniae* infection.

### Immunohistochemistry and TUNEL staining

Paraffin embedded lungs from WT and *Trem-2* ^−/−^ mice were deparaffinized in xylene and ethanol and subjected to antigen-retrieval using citrate buffer pH 6.0 (Vector laboratories). Thereafter endogenous peroxidase activity was blocked with 1.6% H_2_O_2_ in PBS. Following washing and blocking steps using 10% swine or rabbit serum (Vector laboratories) in PBS for the active caspase 3 (Cell signaling) and Ly6G (BD Biosciences) stains respectively, sections were incubated either overnight at 4°C or 1 h room temperature with the active caspase 3 diluted to 1∶25 in swine serum overnight and the Ly6G antibody diluted 1 in 50 in rabbit serum. After washing, endogenous biotin and avidin sites were blocked using the avidin biotin blocking kit (Vector laboratories). Sections were washed, and incubated with either biotinylated anti-rat IgG (Vector Laboratories) or biotinylated, swine anti-rabbit IgG (Dako) for the Ly6G and active caspase 3 stains respectively. Binding was visualized using the Vectastain ABC kit (Vector laboratories) followed by either a step where the sections were incubated with DAB conjugated to HRP (active caspase 3) or Nova Red stain (Vector laboratories, Ly6G stain). Sections were countered with hematoxylin, dehydrated and subjected to light microscopy.

Tunnel stain was performed using the in situ cell death detection kit, according to the manufacturer's instructions (Roche 1684809). Briefly, paraffin embedded lungs from WT and *Trem-2* ^−/−^ mice were deparaffinized in xylene and ethanol and subjected to antigen-retrieval using Pronase E (Sigma). Following washing and incubation with Tunnel reaction mix, nuclei were stained using DAPI (Sigma). Slides were visualized under fluorescence illumination (Zeiss, AxioImager.M2).

### Cell isolation, culture and stimulations

AM were obtained by BAL from healthy WT or *Trem-2* ^−/−^ mice. Cells were resuspended in RPMI 1640 containing 1% penicillin/streptomycin (pen/strep) and 10% FCS and plated for either phagocytosis assays or stimulations at the appropriate density. In some cases this was on C1q (Sigma-Aldrich) coated or BSA coated plates and cells were left to adhere for 3 h or overnight before stimulations. BMDMs were retrieved from the tibia and the femur of mice and differentiated in RPMI 1640 supplemented with 1% pen/strep, 10% FCS and 10% L929-conditioned medium for 7 days.

Primary lung epithelial cells were isolated as previously described [69] and were cultured in in HITES media as were MLE-12 and 15 cells as previously described [31]. RAW 264.7 cells cultured in RPMI 1640 containing 1% pen/strep and 10% FCS. In all experiments related to cytokine production cells were stimulated with 2×10^7^ CFU/ml *S. pneumoniae* (MOI 100) or 10 µg/ml LTA for 6 h unless otherwise indicated.

### Generation of TREM-2 over expressing RAW 264.7 cells

Retroviral transfection was used to generate RAW264.7 cells that were stably transfected with GFP or TREM-2. Briefly, the packaging cell line GP-293 HEK (Clontech) was transfected with TREM-2 expression plasmid (pORF-mTREM-2 originally purchased from InvivoGen) or GFP control plasmids and VSV-G (retroviral vector). RAW 264.7 cells were infected with the virus containing supernatants from HEK cells and successfully transfected, GFP expressing cells were sorted by flow cytometry.

### Luciferase assays

For reporter gene assays, HEK cells seeded at 1.5×10^5^cells/ml, were transfected with PPRE luciferase promoter constructs (provided by Nikolina Papac, Medical University of Vienna), expression vectors encoding PPAR-δ, RXR-α (provided by Ajay Chawla, University of California, San Francisco), vector control (pIRES, Stratagene) or a combination of PPAR-δ, RXR-α together with TREM-2 and DAP-12 (subcloned into the pIRES backbone) using calcium phosphate. 24 h post transfection, cells were stimulated with 1 µM GW0742 and 48 h post transfection luciferase activity was determined using the Dual-Luciferase Reporter Assay System according to the manufacturer's instructions (Promega). All transfectants contained the pRenilla luciferase gene vector (Promega) as an internal control for transfection efficiency and luciferase values were normalized to renilla.

### Preparation of FITC labeled bacteria and CFSE labeled apoptotic cells

*S. pneumoniae* (ATCC 6303 and serotype 19A) or *E.coli* (018:K1) were grown in Todd-Hewitt broth (Difco) or Luria Bertani medium respectively, washed twice and resuspended in saline at a concentration in the range of 10^9^ CFU/ml as determined by OD_600_. Bacteria were heat killed at 65°C, for 30 min, washed once with 10 ml 0.1M NaHCO_3_, resuspended in a solution containing 0.2 mg/ml FITC dissolved in 0.1M NaHCO_3_ and incubated under constant stirring in the dark at 37°C for 1 h. Bacteria were washed twice with PBS and the concentration was set to obtain 2×10^9^ CFU/ml.

Thymocytes were isolated from 4 week old C57BL/6 female mice and apoptosis was induced by culturing 6×10^6^ freshly isolated thymocytes in RPMI/10% FCS with 1 µM dexamethasone for 16 h as previously described [70]. Apoptotic laddering was assayed using a commercially available kit (Roche Diagnostics). 7-AAD/Annexin V staining was performed by resuspending the thymocytes in staining buffer (0.1M Hepes, 1.4M NaCl, 25 mM CaCl_2_, pH 7.4), after which Annexin V (BD Biosciences) was added at 1∶20 for 15 min, following which 7-AAD (eBioscience) was added at 1∶50 for 15 min. Cells were analyzed by flow cytometry (BD LSR Fortessa). Following apoptosis, thymocytes were labeled with CFSE, according to the manufacturer's instructions (Molecular Probes).

### Phagocytosis and efferocytosis assays

AM or BMDM from WT and *Trem-2* ^−/−^ mice were plated at 0.5×10^6^/ml in 12-well microtiter plates (Greiner) and allowed to adhere overnight. After washing steps, FITC-labeled heat-killed *S. pneumoniae* or *E. coli* (O18:K1) was added in the presence of RPMI for 1 h (MOI 100) at 37°C or 4°C (as a negative control), respectively. Cells were treated with proteinase K at 50 µg/ml for 15 min at room temperature to remove adherent but not internalized bacteria and subsequently placed on ice and washed. In some experiments, bacteria were pre-opsonised with either 10% pooled WT mouse serum, C1qa^−/−^ serum [47] or 10% Type III capsular antibody (Statens Serum Institute, Denmark) in RPMI for 30 min before addition to the cells. Uptake was analyzed using a flow cytometer (Beckton Dickinson FACScalibur). The phagocytosis index of each sample was calculated: (mean fluorescence x % positive cells at 37°C) minus (mean fluorescence x % positive cells at 4°C).

Verification of phagocytosis results obtained via FACS was conducted using confocal microscopy as previously described [68]. Briefly, AM plated at 3×10^5^ in 8 well chamberslides (Lab-Tek Chamberslide system) were incubated with FITC-labeled heat-killed *S. pneumoniae* at a MOI 100 for 1 h at 37°C. After washing steps, lysosomes were stained with Lysotracker red and nuclei with DAPI (Invitrogen), followed by visualization using confocal laser scanning microscopy (LSM 510, Zeiss). The ratio of engulfed bacteria (as determined by overlay of green bacteria and red lysosomes) was quantified by an independent researcher from 300 counted cells per well and is expressed as percentage of cells that contain bacteria.

For the in vivo phagocytosis assays WT and *Trem-2* ^−/−^ mice were inoculated intranasally with 5×10^6^ CFU (MOI 10, assuming 5×10^5^ cells in a naïve mouse) FITC-labeled *S. pneumoniae*. BALF was collected 4 h later, cells were resuspended in a PBS supplemented with 1% FCS and antibodies against Ly6G, CD11b, F4/80, CD11c and CD45 and incubated for 30 min. After a washing step, cells were resuspended in PBS and analyzed by flow cytometry (BD LSR Fortessa). AMs were identified as F4/80^+^, CD11c^+^, Ly6G^−^, CD11b^−^ cells. Neutrophils were identified as the Ly6G^+^, CD11b^+^, F4/80^−^ and CD11c^−^ population. To control for background FITC signals and non-specific binding of bacteria, the % of FITC-positive CD45^−^ cells (i.e. non-phagocytosing cells) was subtracted from the % of FITC-positive CD45^+^ cells (i.e. containing phagocytosing cells).

For efferocytosis assays AM from WT and *Trem-2* ^−/−^ mice were incubated with CFSE labelled apoptotic thymocytes at the indicated MOI for 1 h at 37°C or 4°C (as a negative control), respectively. AM were subsequently removed and stained using APC conjugated CD11c antibody (clone N418, eBioscience). Uptake was analyzed using a flow cytometer (Beckton Dickinson FACScalibur). The efferocytosis index of each sample was calculated: (mean fluorescence x % CD11c^+^ CFSE^+^ cells at 37°C) minus (mean fluorescence x % CD11c^+^ CFSE^+^ at 4°C).

### RT-PCR

Trizol was used for RNA extraction from primary cells and cDNA was converted using the Superscript III first strand synthesis system as recommended by the supplier (Invitrogen). RT-PCR was conducted according to the LightCycler FastStart DNA MasterPLUS SYBR Green I system using the Roche Light cycler II sequence detector (Roche Applied Science). Mouse gene-specific primer sequences used are shown in supporting information Table S1. All transcript levels studied were normalized to HPRT.

### Western blotting

5×10^6^ cells were treated as indicated in the figure legends and whole cell extracts were prepared. Cells were washed once with cold PBS, after which the cell pellet was solubilized and lysed in ice cold whole cell extract buffer (20 mM Hepes pH 7.6, 400 mM NaCl, 1 mM EDTA, 5 mM NaF, 500 µM Na_3_VO_4_, 25% glycerol, 0.1% NP-40, 1 mM PMSF, 1 mM DTT, 0.1 mg/ml aprotonin). Lysates were centrifuged at 14,000 rpm for 15 minutes and stored at −80°C. Equal amounts of protein were separated by electrophoresis on a 10% SDS polyacrylamide gel and transferred to polyvinylidene difluoride (PVDF) membranes. Antibodies specific for TREM-2 and PPAR-δ were used at 1∶1000 and β-actin at 1∶500. Immunoreactive proteins were detected by enhanced chemiluminescent protocol (GE Healthcare).

### PPAR-δ activity assay

5×10^6^ cells were treated as indicated in the figure legends and nuclear extracts were prepared by washing the cells with ice cold PBS, followed by three washes in 1 ml of ice cold hypotonic buffer (10 mM HEPES pH 7.9, 1.5 mM MgCl2, 10 mM KCl) to induce swelling. Thereafter, the pellet was resuspended in hypotonic buffer supplemented with 0.1% Nonidet P-40 and incubated on ice for 5 minutes to release nuclei. Subsequently, samples were centrifuged at 4°C to pellet nuclei, the cytoplasmic fraction was removed and the nuclear pellett resuspended in 50 µl of ice cold high salt buffer (20 mM HEPES pH 7.9, 1.5 mM MgCl2, 420 mM NaCl, 25% glycerol) and incubated for 15 minutes at 4°C. Disrupted nuclei were centrifuged at full speed in a table top centrifuge for 15 minutes at 4°C, after which the supernatant (nuclear fraction) removed. PPAR-δ DNA binding activity in nuclear extracts was determined using the PPAR-δ transcription factor assay kit according to the manufacturer's instructions (Abcam). In brief, nuclear extracts were incubated on a 96 well plate to which the PPRE consensus sequence was immobilized. After binding and washing, PPAR-δ activity within nuclear extracts was detected using a PPAR-δ specific antibody followed by incubation with a secondary HRP conjugated antibody and quantification using spectrophotometry. The specific OD was calculated by subtracting the non specific binding wells (i.e. wells where nuclear extract was absent), and normalized to protein content.

### Microarray

Isolated total RNA was purified using the RNeasy kit per manufacturer's instructions (Qiagen). Total RNA (200 ng) was then used for GeneChip analysis. Preparation of terminal-labeled cDNA, hybridization to genome-wide murine GeneLevel 1.0 ST GeneChips (Affymetrix), and scanning of the arrays were carried out according to manufacturer's protocols. Affymetrix microarray cell intensity files were combined, and expression was normalized using the robust multi-array average algorithm [71], generating an expression matrix. Identification of differentially regulated genes was performed with significance analysis of microarrays as described previously [72]; a false-discovery rate of 5% was imposed. All data are deposited at Gene Expression Omnibus (http://www.ncbi.nlm.nih.gov/geo/) and the accession ID is GSE51378.

### Statistical analysis

Data are presented as the mean ± SEM. Comparisons between groups was assessed using either T-test or ANOVA followed by Tukey's multiple comparisons analysis, where appropriate. Survival data was analyzed by Log rank (Mantel-Cox) test using GraphPad Prism Software.

## Supporting Information

Figure S1 **TREM-2 deficient AM exhibit enhanced phagocytosis of** ***S. pneumoniae*** **as determined by confocal microscopy.** WT and *Trem-2* ^−/−^ AM were incubated with FITC labeled *S. pneumoniae* at an MOI of 100 and confocal microscopy was conducted as described in the materials and methods. Depicted are the original pictures. Magnification ×100.
(PDF)

Figure S2 **TREM-2 deficient BMDM exhibit lower phagocytosis of** ***E.coli*** **.** WT and *Trem-2* ^−/−^ BMDM (n = 5 per genotype) were incubated with FITC labeled *E. coli* at an MOI of 100 and phagocytosis was assessed 1 h later by FACS. Data are presented as mean ± SEM versus WT, **** p<0.0001 and are representative of two independent experiments.
(PDF)

Figure S3 **No difference in surface expression of the phagocytic receptors CD36 and MARCO between WT and TREM-2^−/−^ AM.** Basal surface expression of CD36 and MARCO in WT versus *Trem-2* ^−/−^ AMs as determined by FACS. Green lines depict WT macrophages, pink *Trem-2* ^−/−^ macrophages and black represents isotype control antibody.
(PDF)

Figure S4 **Overexpression of TREM-2 lowers** ***C1qc*** **levels.** *C1qc* basal levels were determined in GFP control or TREM-2 overexpressing RAW 264.7 macrophages using RT-PCR (n = 4 per condition). Data are presented as mean ± SEM and are representative of two independent experiments, *** indicates p<0.001.
(PDF)

Figure S5 **Dependence of C1q expression in AMs on PPAR-δ.** (**A**) WT AM (n = 3–4 per condition) were pre-treated for 24 h with the indicated doses of the PPAR-δ activator GW0742 in combination with the PPAR-δ inhibitor GSK3787 along with a DMSO control after which RT-PCR of *C1qb* and *C1qc* was performed. (**B**) WT and *Trem-2* ^−/−^ AM (n = 4 per condition) were treated with DMSO or the PPAR-δ inhibitor GSK3787 after which RT-PCR of *C1qb* and *C1qc* was performed. Data are presented as mean ± SEM and compared versus WT with * p<0.05, ** p<0.005.
(PDF)

Figure S6 **No difference in PPAR-δ levels between WT and** ***TREM-2^−/−^*** **AM.** (**A**) *PPAR-δ* basal expression was determined in WT and *Trem-2* ^−/−^ AM using RT-PCR (n = 3–4 per genotype). (**B**) PPAR-δ protein levels from whole cell extracts of WT and *Trem-2* ^−/−^ AM. The specificity of the antibody was indicated by recombinant PPAR-δ (rPPAR-δ). Data are representative of two independent experiments.
(PDF)

Figure S7 **Unaltered C3a and C5a levels in the BALF of** ***TREM-2^−/−^*** **mice following** ***S. pneumoniae*** **infection.** WT and *Trem-2* ^−/−^ mice (n = 6–7 mice per genotype) were intranasally infected with 10^5^ *S. pneumoniae* and C3a and C5a levels were determined in the BALF 6 and 24 h post infection. Data represent mean ± SEM versus WT.
(PDF)

Figure S8 **Specificity of antibodies used in Immunohistochemistry and TUNEL.** Representative Ly6G, active caspase 3, and TUNEL staining of lungs 48 h post infection of mice infected with 10^5^ *S. pneumoniae* (n = 9) depicting the specificities of the antibodies used.
(PDF)

Table S1 **Sequences of primers used for RT-PCR.**
(DOCX)
