# Supplementary material for: The Triggering Receptor Expressed on Myeloid Cells 2 Inhibits Complement Component 1q Effector Mechanisms and Exerts Detrimental Effects during Pneumococcal Pneumonia
Source: PLoS Pathog. 2014 Jun 12;10(6):e1004167. doi: 10.1371/journal.ppat.1004167 (PMC4055749; doi:10.1371/journal.ppat.1004167)
Supplement: Figure S1 — TREM-2 deficient AM exhibit enhanced phagocytosis of S. pneumoniae as determined by confocal microscopy. WT and Trem-2 −/− AM were incubated with FITC labeled S. pneumoniae at an MOI of 100 and confocal microscopy was conducted as described in the materials and methods. Depicted are the original pictures. Magnification ×100. (PDF) [file ppat.1004167.s001.pdf]

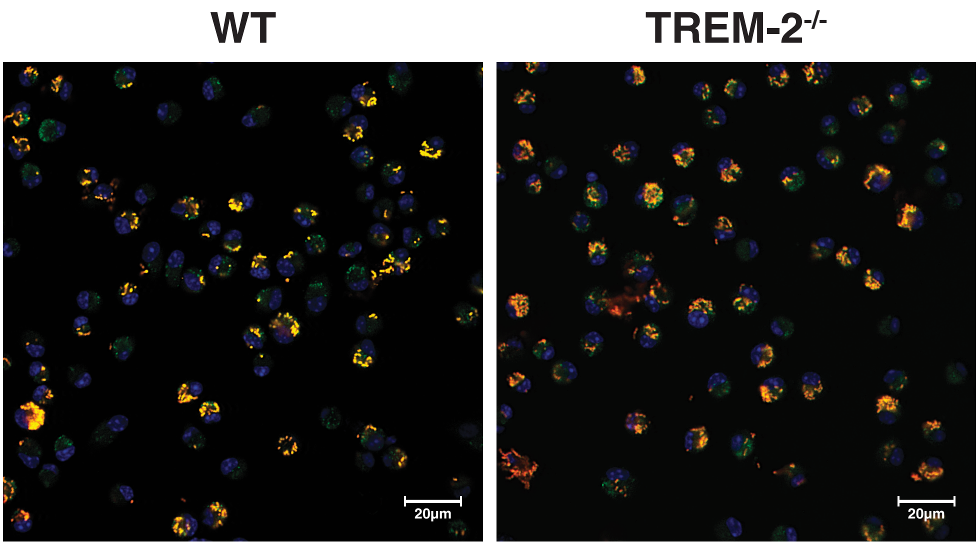

Supplementary Figure 1: TREM-2 deficient AM exhibit enhanced phagocytosis of *S. pneumoniae* as determined using confocal microscopy

WT and *Trem-2*$^{-/-}$ AM were incubated with FITC labeled *S. pneumoniae* at an MOI of 100 and confocal microscopy was conducted as described in the materials and methods. Depicted are the original pictures. Magnification x 100.
