# Supplementary material for: The Triggering Receptor Expressed on Myeloid Cells 2 Inhibits Complement Component 1q Effector Mechanisms and Exerts Detrimental Effects during Pneumococcal Pneumonia
Source: PLoS Pathog. 2014 Jun 12;10(6):e1004167. doi: 10.1371/journal.ppat.1004167 (PMC4055749; doi:10.1371/journal.ppat.1004167)
Supplement: Figure S2 — TREM-2 deficient BMDM exhibit lower phagocytosis of E.coli . WT and Trem-2 −/− BMDM (n = 5 per genotype) were incubated with FITC labeled E. coli at an MOI of 100 and phagocytosis was assessed 1 h later by FACS. Data are presented as mean ± SEM versus WT, **** p<0.0001 and are representative of two independent experiments. (PDF) [file ppat.1004167.s002.pdf]

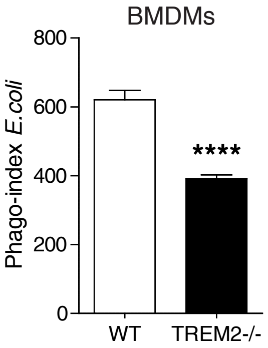

Supplementary Figure 2: TREM-2 deficient BMDM exhibit lower phagocytosis of *E.coli*

WT and *Trem-2*$^{-/-}$ BMDM were incubated with FITC labeled *E. coli* at an MOI of 100 and phagocytosis was assessed 1h later by FACS. Data are presented as mean ± SEM versus WT, **** $p < 0.0001$ and are representative of two independent experiments.
