# Supplementary material for: The Triggering Receptor Expressed on Myeloid Cells 2 Inhibits Complement Component 1q Effector Mechanisms and Exerts Detrimental Effects during Pneumococcal Pneumonia
Source: PLoS Pathog. 2014 Jun 12;10(6):e1004167. doi: 10.1371/journal.ppat.1004167 (PMC4055749; doi:10.1371/journal.ppat.1004167)
Supplement: Figure S3 — No difference in surface expression of the phagocytic receptors CD36 and MARCO between WT and TREM-2−/− AM. Basal surface expression of CD36 and MARCO in WT versus Trem-2 −/− AMs as determined by FACS. Green lines depict WT macrophages, pink Trem-2 −/− macrophages and black represents isotype control antibody. (PDF) [file ppat.1004167.s003.pdf]

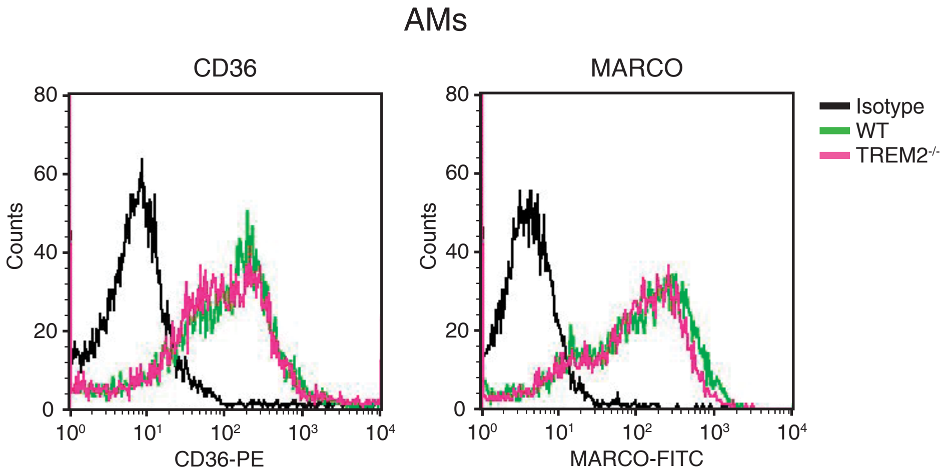

Supplementary Figure 3: No difference in surface expression of the phagocytic receptors CD36 and MARCO between WT and and *TREM-2$^{-/-}$* AM

Basal surface expression of CD36 and MARCO in WT versus *Trem-2$^{-/-}$* AMs as determined by FACS. Green lines depict WT macrophages, pink *Trem-2$^{-/-}$* macrophages and black represents isotype control antibody.
