# Supplementary material for: The Triggering Receptor Expressed on Myeloid Cells 2 Inhibits Complement Component 1q Effector Mechanisms and Exerts Detrimental Effects during Pneumococcal Pneumonia
Source: PLoS Pathog. 2014 Jun 12;10(6):e1004167. doi: 10.1371/journal.ppat.1004167 (PMC4055749; doi:10.1371/journal.ppat.1004167)
Supplement: Figure S4 — Overexpression of TREM-2 lowers C1qc levels. C1qc basal levels were determined in GFP control or TREM-2 overexpressing RAW 264.7 macrophages using RT-PCR (n = 4 per condition). Data are presented as mean ± SEM and are representative of two independent experiments, *** indicates p<0.001. (PDF) [file ppat.1004167.s004.pdf]

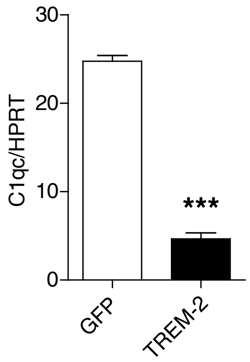

Supplementary Figure 4: Overexpression of TREM-2 lowers *C1qc* levels

C1qc basal levels were determined in GFP control or TREM-2 over expressing RAW 264.7 macrophages using RT-PCR. Data are presented as mean ± SEM and are representative of two independent experiments, *** indicates $p < 0.001$.
