# Supplementary material for: The Triggering Receptor Expressed on Myeloid Cells 2 Inhibits Complement Component 1q Effector Mechanisms and Exerts Detrimental Effects during Pneumococcal Pneumonia
Source: PLoS Pathog. 2014 Jun 12;10(6):e1004167. doi: 10.1371/journal.ppat.1004167 (PMC4055749; doi:10.1371/journal.ppat.1004167)
Supplement: Figure S5 — Dependence of C1q expression in AMs on PPAR-δ. (A) WT AM (n = 3–4 per condition) were pre-treated for 24 h with the indicated doses of the PPAR-δ activator GW0742 in combination with the PPAR-δ inhibitor GSK3787 along with a DMSO control after which RT-PCR of C1qb and C1qc was performed. (B) WT and Trem-2 −/− AM (n = 4 per condition) were treated with DMSO or the PPAR-δ inhibitor GSK3787 after which RT-PCR of C1qb and C1qc was performed. Data are presented as mean ± SEM and compared versus WT with * p<0.05, ** p<0.005. (PDF) [file ppat.1004167.s005.pdf]

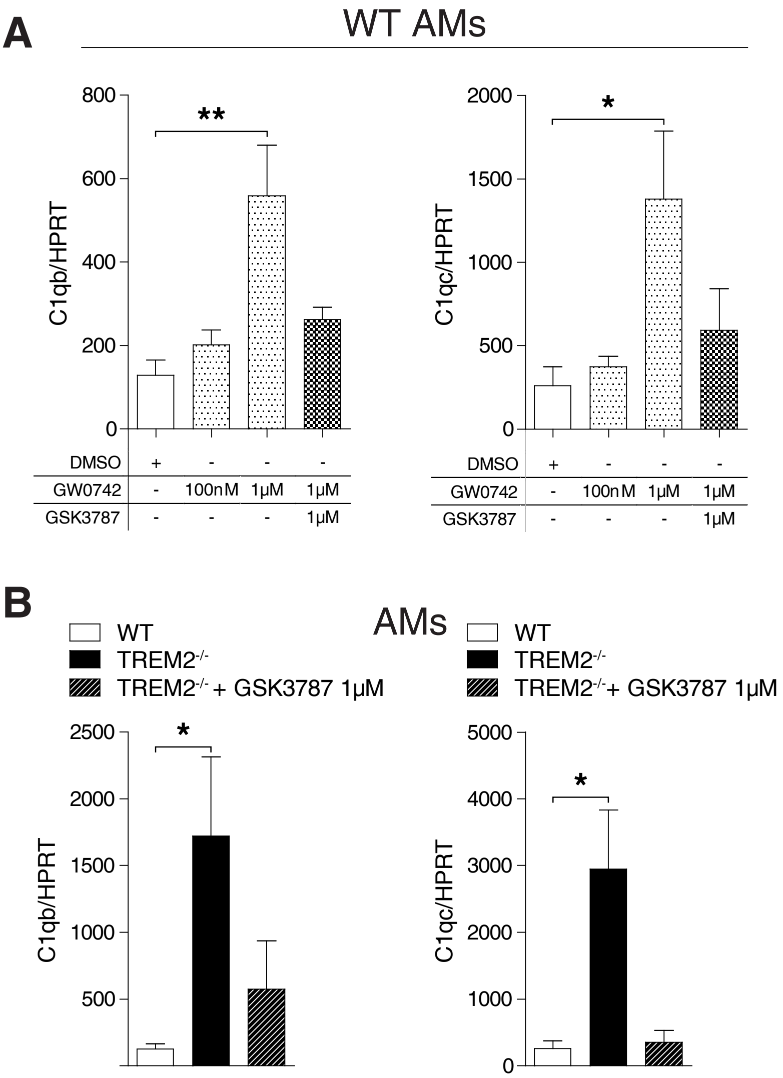

Supplementary Figure 5: Dependence of C1q expression in AMs on PPAR-δ

(A) WT AM were pre-treated for 24h with the indicated doses of the PPAR-δ activator GW0742 in combination with the PPAR-δ inhibitor GSK3787 along with a DMSO control after which RT-PCR of *C1qb* and *C1qc* was performed. **(B)** WT and *Trem-2*$^{-/-}$ AM were treated with DMSO or the PPAR-δ inhibitor GSK3787 after which RT-PCR of *C1qb* and *C1qc* was performed. Data are presented as mean ± SEM and compared versus WT with * $p < 0.05$, ** $p < 0.005$.
