# Supplementary material for: The Triggering Receptor Expressed on Myeloid Cells 2 Inhibits Complement Component 1q Effector Mechanisms and Exerts Detrimental Effects during Pneumococcal Pneumonia
Source: PLoS Pathog. 2014 Jun 12;10(6):e1004167. doi: 10.1371/journal.ppat.1004167 (PMC4055749; doi:10.1371/journal.ppat.1004167)
Supplement: Figure S6 — No difference in PPAR-δ levels between WT and TREM-2−/− AM. (A) PPAR-δ basal expression was determined in WT and Trem-2 −/− AM using RT-PCR (n = 3–4 per genotype). (B) PPAR-δ protein levels from whole cell extracts of WT and Trem-2 −/− AM. The specificity of the antibody was indicated by recombinant PPAR-δ (rPPAR-δ). Data are representative of two independent experiments. (PDF) [file ppat.1004167.s006.pdf]

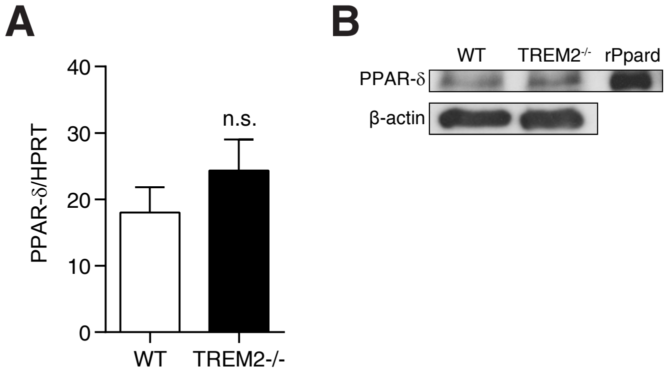

Supplementary Figure 6: No difference in PPAR-δ levels between WT and *TREM-2$^{-/-}$* AM

(A) *PPAR-δ* basal expression was determined in WT and *Trem-2$^{-/-}$* AM using RT-PCR (n = 3-4 per genotype). (B) PPAR-δ protein levels from whole cell extracts of WT and TREM2$^{-/-}$ AM. The specificity of the antibody was indicated by recombinant PPAR-δ (rPPAR-δ). Data ara representative of two independent experiments.
