# Supplementary material for: The Triggering Receptor Expressed on Myeloid Cells 2 Inhibits Complement Component 1q Effector Mechanisms and Exerts Detrimental Effects during Pneumococcal Pneumonia
Source: PLoS Pathog. 2014 Jun 12;10(6):e1004167. doi: 10.1371/journal.ppat.1004167 (PMC4055749; doi:10.1371/journal.ppat.1004167)
Supplement: Figure S7 — Unaltered C3a and C5a levels in the BALF of TREM-2−/− mice following S. pneumoniae infection. WT and Trem-2 −/− mice (n = 6–7 mice per genotype) were intranasally infected with 105 S. pneumoniae and C3a and C5a levels were determined in the BALF 6 and 24 h post infection. Data represent mean ± SEM versus WT. (PDF) [file ppat.1004167.s007.pdf]

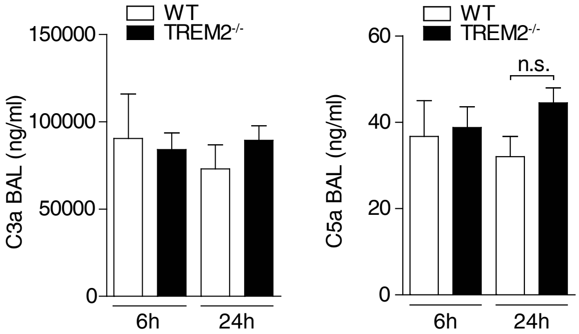

Supplementary Figure 7: Unaltered C3a and C5a levels in the BALF of *TREM-2$^{-/-}$* mice following *S. pneumoniae* infection

WT and *Trem-2$^{-/-}$* mice (n = 6-7 mice per genotype) were intranasally infected with 10^5 *S. pneumoniae* and C3a and C5a levels were determined in the BALF 6 and 24h post infection. Data represent mean ± SEM versus WT.
