# Supplementary material for: The Triggering Receptor Expressed on Myeloid Cells 2 Inhibits Complement Component 1q Effector Mechanisms and Exerts Detrimental Effects during Pneumococcal Pneumonia
Source: PLoS Pathog. 2014 Jun 12;10(6):e1004167. doi: 10.1371/journal.ppat.1004167 (PMC4055749; doi:10.1371/journal.ppat.1004167)
Supplement: Figure S8 — Specificity of antibodies used in Immunohistochemistry and TUNEL. Representative Ly6G, active caspase 3, and TUNEL staining of lungs 48 h post infection of mice infected with 105 S. pneumoniae (n = 9) depicting the specificities of the antibodies used. (PDF) [file ppat.1004167.s008.pdf]

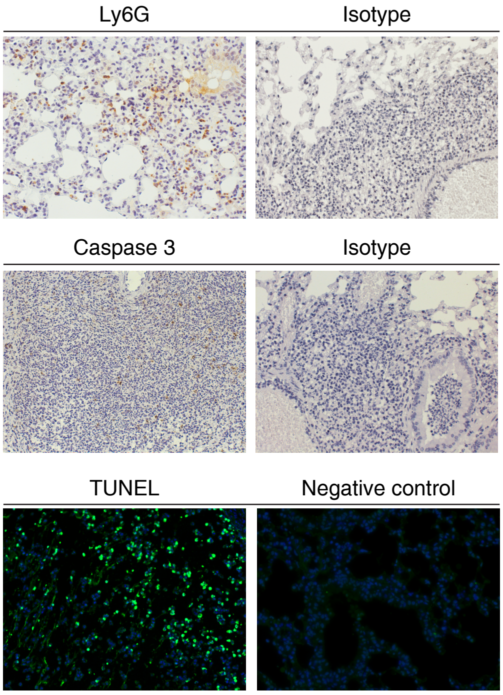

Supplementary Figure 8: Specificity of antibodies used in Immunohistochemistry and TUNEL

Representative Ly6G, active caspase 3, and TUNEL staining of lungs 48h post infection of mice infected with 10^5 *S. pneumoniae* (n= 9) depicting the specificites of the antibodies used.
