# Supplementary material for: The Triggering Receptor Expressed on Myeloid Cells 2 Inhibits Complement Component 1q Effector Mechanisms and Exerts Detrimental Effects during Pneumococcal Pneumonia
Source: PLoS Pathog. 2014 Jun 12;10(6):e1004167. doi: 10.1371/journal.ppat.1004167 (PMC4055749; doi:10.1371/journal.ppat.1004167)
Supplement: Table S1 — Sequences of primers used for RT-PCR. (DOCX) [file ppat.1004167.s009.docx]

**Supporting information Table 1**

Sequences of primers used for RT-PCR

| **Gene** | **5‘** | **3‘** |
| --- | --- | --- |
| C1qb | GCAGCAGGCTCTGGGCTCTGGGA | ATGCCAGGGGGCCCGGTGCA |
| C1qc | CTGTCTGGGAGAACAGGACGTCTCT | GGCATGCCAGGCTCGCCCTT |
| THBS1 | CCCCGGTGCACACAGGCTCCG | TCGGCGACCGGGGCCCCTTC |
| TREM-1 | ATGACCTAGTGGAGGGCCAG | GCACAACAGGGTCATTCGGAG |
| TREM-2 | TTGCTGGAACCGTCACCATC | CACTTGGGCACCCTCGAAAC |
| HPRT | GTTAAGCAGTACAGCCCCAAAATG | AAATCCAACAAAGTCTGGCCTGTA |
